# Stability evaluation of compounded clonidine hydrochloride oral liquids based on a solid-phase extraction HPLC-UV method

**DOI:** 10.1371/journal.pone.0260279

**Published:** 2021-11-30

**Authors:** Daphné Coache, Mihaela Friciu, V. Gaëlle Roullin, Marianne Boulé, Jean-Marc Forest, Grégoire Leclair

**Affiliations:** 1 Faculty of Pharmacy, Université de Montréal, Montréal, Québec, Canada; 2 Sainte-Justine University Hospital Center, Montréal, Québec, Canada; Laurentian University, CANADA

## Abstract

The present study aimed to assess the stability of clonidine hydrochloride oral liquids (20-μg/mL) prepared from two different generic tablets in Ora-Blend and stored in amber plastic bottles. Physical and chemical stabilities were evaluated over a period of 90 days at 25°C. Analytical challenges were overcome with the development of a new extraction procedure based on solid phase extraction to ensure efficient clonidine hydrochloride quantification. The absence of physical instabilities, evaluated by qualitative and quantitative measurements (static multiple light scattering), as well as the absence of chemical instabilities, evidenced by a stability-indicating HPLC-UV method, confirmed that a beyond-use date of 90 days was appropriate for these compounded oral liquids.

## Introduction

Clonidine hydrochloride, an α-2 adrenergic receptor agonist, is indicated for the treatment of hypertension in adults [1]. Even though clonidine hydrochloride has only been approved by Health Canada for its effects on cardiac output and blood pressure in adults, off-label use in children is not unusual. For inpatient, clonidine hydrochloride can be used for multiple indications, like neonatal abstinence syndrome, analgesia, sedation, and hypertensive crisis [2]. A slow tapering of clonidine is sometimes needed, for example, in the treatment of neonatal abstinence syndrome or after a long term use of dexmedetomidine, which is also an α-2 adrenergic receptor agonist with sedative properties used in intensive care unit [3]. Therefore, it is really important to have access to a compounded clonidine formulation that will allow a slow tapering of the dose adapted to the pediatric population. Clonidine hydrochloride can also be prescribed for chronic health problem, like deficit/hyperactivity disorder, insomnia, tic disorders and spasticity for example [4–8]. A compounded formulation with longer stability is thus needed for practical reasons for pediatric outpatient. Even if tablets of variable strengths are commercially available in Canada (25, 100 and 200 μg), liquid formulations must be compounded by pharmacists since tablets do not allow a precise dose according to the patient weight.

The stability of clonidine hydrochloride has been studied in a large variety of oral vehicles at different concentrations: 10 μg/mL in Ora-Blend, Oral-Mix and Oral-Mix SF [9, 10] as well as 20 μg/mL and 100 μg/mL in simple syrup [11, 12]. Oral liquids displaying a concentration of 100 μg/mL are inappropriate for neonates, for whom lower concentrations are required considering the low doses prescribed. For example, if a prescription of 5 μg is needed, 0.05 mL should be dispensed, which is quite difficult using a syringe for oral use. Therefore, concentrations of 10 and 20 μg/mL are more appropriate for small doses and will result in a more precise administration. Indeed, clonidine hydrochloride is a medication with high risk of dosing errors in children and several cases of overdose have been reported, some of which are related to compounding errors [13–16]. For instance, a tenfold higher concentration, such as 100 μg/mL instead of 10 μg/mL, is a possible source of error when both formulation compounding files coexist in the same workplace [17]. This risk is even greater when the concentration is reported in mg/mL where an error of decimal, for example 0.01 mg/mL instead of 0.1 mg/mL would dramatically impact the safety of the delivered preparation [18]. On the contrary, the co-existence of 100- and 20-μg/mL compounding files would generate less confusion. Moreover, a 20-μg/mL preparation remains a versatile concentration which could be used both for adult and children prescriptions. These are the two main reasons why 20 μg/mL seems to be the most interesting concentration for the development of a new formulation.

The choice of vehicle is a crucial factor to consider to compound a clonidine hydrochloride suspension. When oral suspensions are prepared in simple syrup, after only few hours of storage, an important sediment forms at the bottom of the bottle. This sediment is almost impossible to resuspend, even after long minutes of handshaking. This physical instability leads to poor content uniformity, resulting in a lack of safety for patients receiving this formulation. Simple syrup, a widely used compounding vehicle, is therefore not the choice vehicle for such a compounding. In fact, an internal report from the pharmacy department of Ste-Justine Hospital (Montréal, Canada) concluded in 2018 that a 20-μg/mL clonidine hydrochloride suspension in Ora-Blend or Oral-Mix should be developed, in order to replace simple syrup and overcome physical instabilities [18].

According to these observations, the development of a safer and more appropriate formulation is required.

Chemical stability of clonidine hydrochloride (10 μg/mL) has already been evaluated in readily available commercial vehicles, such as Oral-Mix (Medisca, QC, Canada) and Ora-Blend (Galenova, QC, Canada), which make them good candidates for the development of a new formulation at a concentration of 20 μg/mL [9, 10]. Ora-Blend is particularly interesting for pediatric compoundings since this sweetened oral suspending vehicle, widely used to perform extemporaneous compounding of oral suspensions, is well-accepted in children. Moreover, its list of excipients (trisodium phosphate, glycerine, cellulose and sucrose) is well-established and microbiologically sound, which guarantees a very low risk of microorganism growth if compounded in an adequate manner.

Therefore, this article presents the chemical and physical stabilities of 20-μg/mL clonidine hydrochloride suspensions prepared from two generic tablets in the Ora-Blend suspension vehicle, over a period of 90 days at room temperature. To overcome analytical challenges, a new sample preparation method has been developed for the analysis of clonidine hydrochloride, using a stability indicating HPLC-UV method.

## Materials

Acetonitrile, methanol, *o*-phosphoric acid (85%), formic acid and ammonium hydroxide (23%) of HPLC grade as well as hydrogen peroxide solution (H_2_O_2_, 30%), hydrochloric acid solution (HCl, 37%) were purchased from Fisher Scientific (QC, Canada). Sodium hydroxide (NaOH) pellets were purchased from MilliporeSigma (ON, Canada). Trifluoroacetic acid (99%) was purchased from Sigma-Aldrich (ON, Canada). Clonidine hydrochloride powder was purchased from Toronto Research Chemicals (ON, Canada; lot# 10-XJZ-26-1). Clonidine hydrochloride tablets of 100 μg (Teva, lot #35213985A; Mint Pharmaceuticals, lot #CDT1e1L004), Ora-Blend (Galenova, lot #9278160), 60-mL amber polyethylene terephthalate (PET) bottles with child-resistant caps (Richards Packaging, ON, Canada) and an oral liquid prepared from the Teva tablets in simple syrup were graciously provided by the pharmacy department of the Ste-Justine Hospital (QC, Canada).

## Methods

### Preparation of clonidine hydrochloride oral liquids

Clonidine hydrochloride oral liquids (20 μg/mL) were compounded from 100-μg tablets in Ora-Blend. Tablets (20 tablets) were grounded into fine powder with a mortar and a pestle. The powder was then mixed with a small amount of Ora-Blend until a smooth paste was achieved. Additional vehicle (approximatively 70% of the final volume) was added in successive portions to the mortar, while taking care of mixing after each addition. The mixture was transferred in a graduated cylinder. The mortar and pestle were rinsed with two 10-mL portions of Ora-Blend and then the oral liquid was completed to the desired final volume (100 mL). The oral liquids were finally mixed to ensure homogeneity.

### Design of the stability study

Three oral liquids were prepared from both generic tablets. All six oral liquids were packaged in amber plastic (PET) bottles with child-resistant caps and stored at controlled room temperature (25 ± 2°C / 60 ± 5% RH–Thermo Scientific, Forma Environmental Chamber, OH, USA).

At predetermined time points (0, 30, 60 and 90 days), bottles were handshaken (20 s), and the oral liquids were visually evaluated for consistency, color and odor changes. From each bottle, an aliquot (1.0 mL) was sampled and transferred in a 5 mL-centrifuge tubes for further analysis. All samples were kept at -80°C until the end of the study. The pH of all preparations was evaluated on day 0 and 90 using a pH meter (pH 211, Hanna Instruments, Montréal, QC, Canada). Means and standard deviations were calculated from 3 independent experiments and were used to compare formulation behaviours.

### Physical stability evaluation

Additionally, the physical stability of the preparations was assessed with the aid of the Turbiscan LAB Stability Analyzer (Formulaction, Toulouse, France). The advantage of this instrument, based on the static multiple light scattering (SMLS) technology, is to provide quantitative stability data for opaque dispersions and to detect physical instabilities that could not be detected with the naked eye. The principle of this method is based on transmitted and backscattered lights which are repeatedly recorded at various heights of the sample. Signals recorded by both detectors are used for the calculation of the Turbiscan Stability Index (TSI), a measurement of the global physical stability of the analyzed formulation [19, 20]. In this article, TSI values were used for comparison purposes to describe the physical stability of the oral liquids over time, but also to compare different vehicles. Prior to analysis, bottles of oral liquid were handshaken (20 s) to ensure homogeneity and glass vials (25 mL) were immediately filled with the oral liquid and analyzed (20 min, 40 scans, temperature set at 25°C, scanning height: 40 mm).

### HPLC- UV method

The quantification of clonidine hydrochloride was performed using an HPLC system (Shimadzu Prominence UFLC) comprising a binary pump (LC-20AD), a solvent degasser (DGU-20A5), a multiple wavelength photodiode array detector (SPD-M20A), a refrigerated autosampler (SIL020AC HT) and a column oven (CTO-20AC). Integration and peak purity were calculated with the help of the HPLC software (LabSolutions 5.54 sp5, Shimadzu Corporation).

The separation was performed on a Kinetex XB- C18 chromatographic column (3.0 x 100 mm, 5 μm, Phenomenex, Torrance, CA, USA). The column temperature was kept at 40°C and the UV detector wavelength was set at 210 nm. The flow rate was 1 mL/min and the injection volume was 10 μL. The composition of mobile phase A was a mixture of 923.0 mL of purified water, 77.0 mL of acetonitrile and 0.5 mL of trifluoroacetic acid, while mobile phase B was acetonitrile. Between 0 and 4 min, mobile phase A was 100%. Then mobile phase A proportion was reduced to 30% in 0.5 min and remained the same for 1 min. Finally mobile phase A was set back to its initial conditions in 0.5 min, for re-equilibration until 15 min.

### Sample preparation for HPLC injection

**Fig 1:**
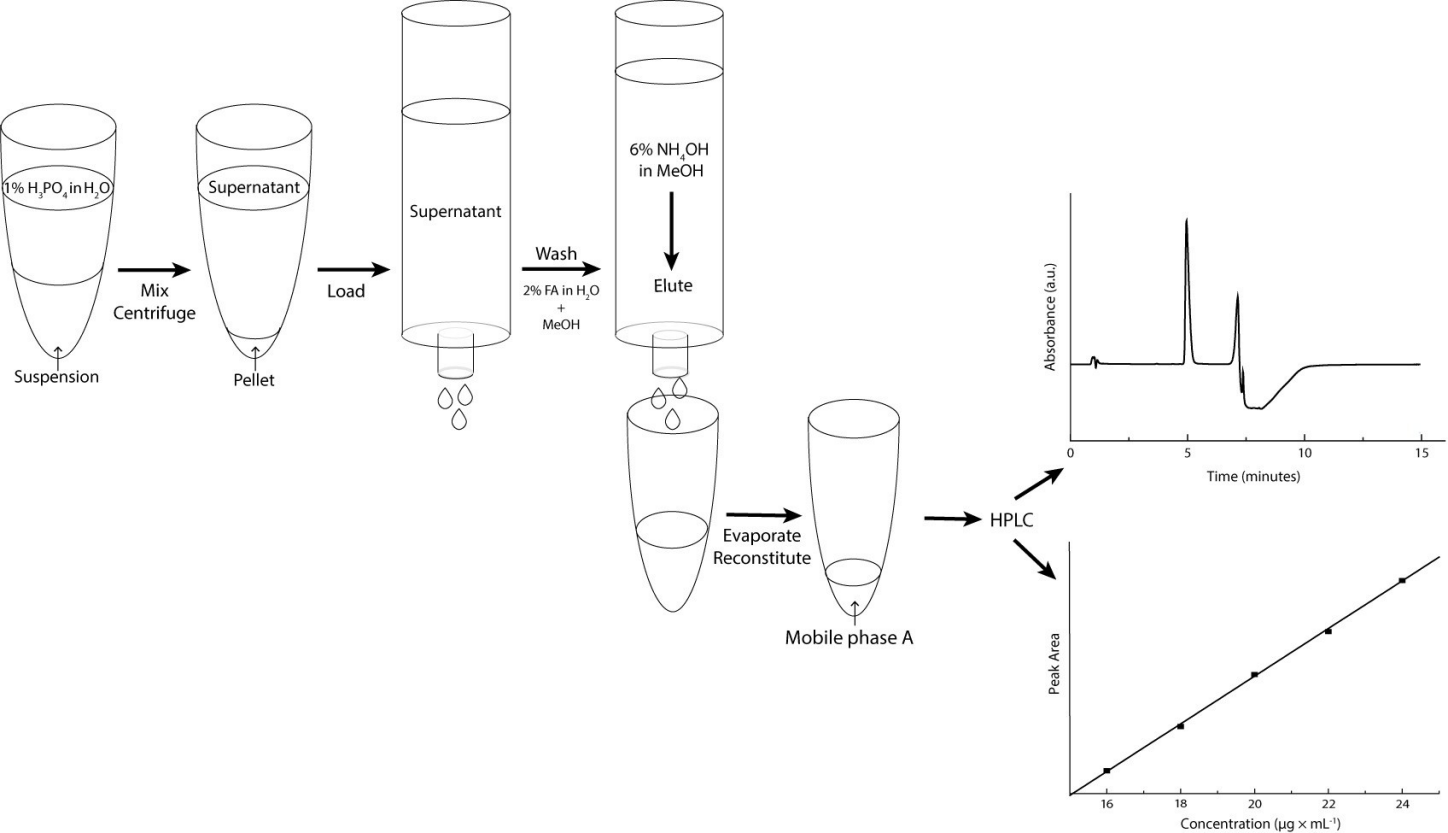
Schematic representation of clonidine hydrochloride sample preparation for analysis of oral liquids using HPLC-UV.

Phosphoric acid 0.1% in water (1.0 mL) was added to the 5 mL-centrifuge tubes containing the clonidine oral liquid samples (1.0 mL). Tubes were vigorously vortexed (20 s) and then centrifuged (2135 g, 15 min). This resulted in a phase separation: part of the vehicle and tablet excipients precipitated. An Oasis MCX SPE cartridge (1 mL/30 mg, Waters, MA, USA) attached to a Cerex 48 positive pressure SPE apparatus (Cerex, GA, USA) was loaded with 800 μL of supernatant. The cartridge was first washed with ammonium formate 100 mM with 2% formic acid in water (1.0 mL) and then with methanol (1.0 mL). Ammonium hydroxide 6% in methanol (1.0 mL) was used for the elution. Eluates were collected in glass tubes and evaporated to dryness in a water bath set at 40°C under a stream of nitrogen gas (TurboVap LV, Biotage, Uppsala, SE). Residues were reconstituted in mobile phase A (200 μL) and injected in duplicate into the HPLC system (Fig 1).

### Calibration curve preparation

A 5-point calibration curve (16, 18, 20, 22, 24 μg/mL) was prepared with clonidine hydrochloride powder in Ora-Blend. This calibration range covered 80.0 to 120.0% of the target concentration. A standard stock solution of 1 mg/mL was used to prepare a 24-μg/mL standard solution. From this standard solution, the remaining concentrations were prepared by serial dilutions. Ora-Blend without clonidine hydrochloride was used as blank. These standard solutions were then extracted following the sample preparation for HPLC injection procedure and injected in triplicate in the HPLC system. Intraday variability was calculated for each concentration using the calibration curve. Interday variability was calculated from the results of the calibration curve injected on three consecutive days.

### Forced degradation study

Specificity of the analytical method clonidine hydrochloride was evaluated by mixing 1.0 mL of the clonidine hydrochloride oral liquid (20 μg/mL) with 250 μL of each of the following solutions: H_2_O, 30% H_2_O_2_, 10 N HCl and 10 N NaOH. All samples were heated at 60°C for at least 4 hours. A reference sample was prepared by mixing 250 μL H_2_O with oral liquid and kept refrigerated for the duration of the incubation. After that, samples were brought back to room temperature and 250 μL of water were added to the reference, H_2_O and H_2_O_2_, while HCl and NaOH were neutralized with the same volume of 10 N NaOH and 10 N HCl, respectively. Sample preparation for HPLC injection was then performed as abovementioned, prior to the HPLC-UV analysis.

### Specifications

To be considered acceptable, the remaining percentage of the initial concentration, calculated with a stability-indicating HPLC-UV method, should be not less than 90.0% or not more than 110.0% at each time points. A change of less than one pH unit was considered acceptable, otherwise, investigation should be carried out in order to explain this variation.

## Results

### Physical stability assessment

Physical stability was first evaluated visually. Observations were the same for both generic tablets. Both oral liquids were less viscous than the one prepared in simple syrup, and no crystallisation or caking was observed over the entire study period. Between analysis time points, separation of bottle content was observed but gentle hand shaking of a few seconds was enough to ensure complete homogenous resuspension. The color of the oral liquids did not change over time: the oral liquids prepared from Mint and Teva tablets were pinkish and whitish, respectively.

**Fig 2:**
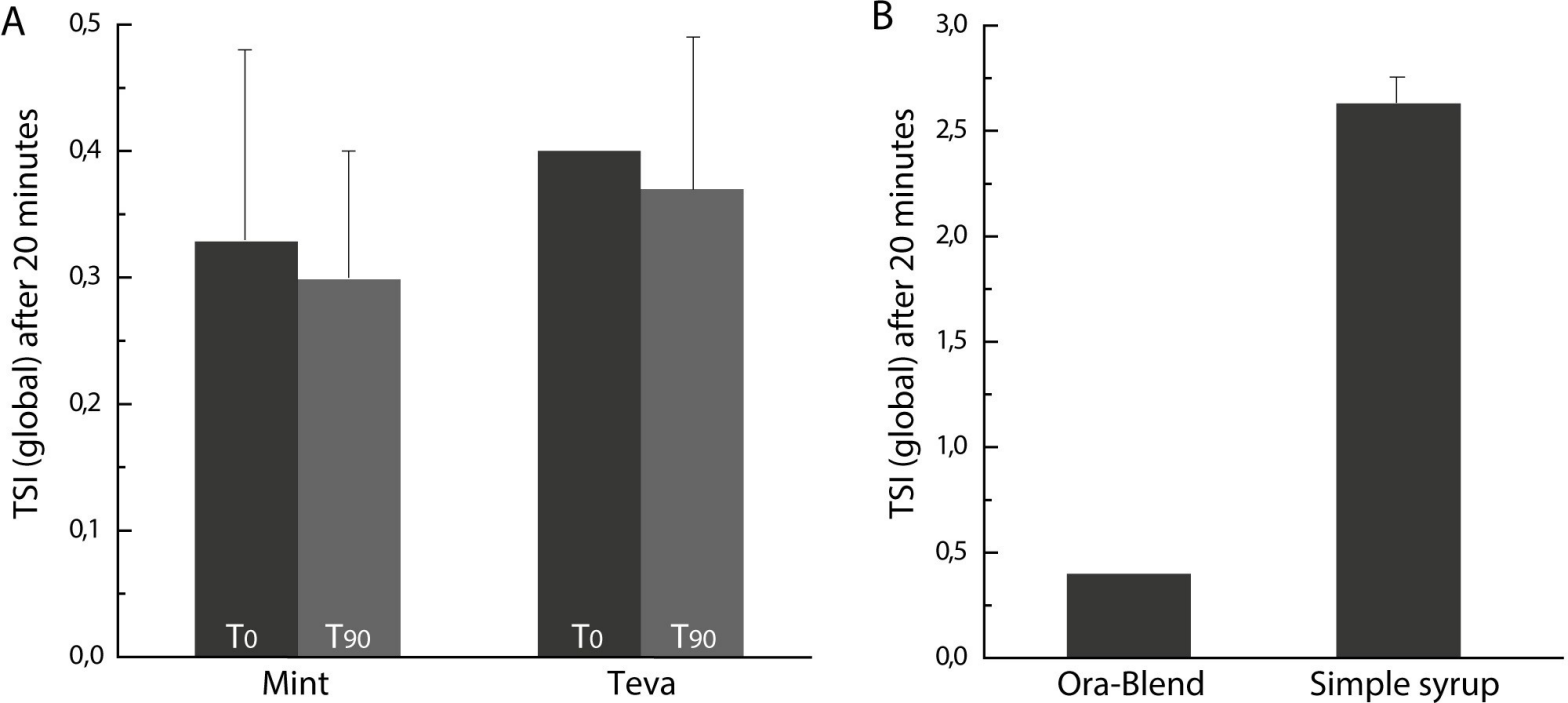
TSI measurements after a 20-min stability analysis. (A) oral liquids compounded from two generic tablets on day 0 and 90 and resuspended 20 s by gentle hand shaking; (B) for oral liquids prepared from tablets (Teva) in Ora-Blend or simple syrup on the day of preparation. Mean ± SD (correspond to standard deviation of measurements from three different bottles).

Further analysis of the physical stability was performed using Turbiscan Stability Analyzer. The TSI calculated at the beginning and the end of the study for the oral liquids prepared from both tablets in Ora-Blend were similar (Fig 2A). For comparison purposes, an oral liquid prepared in simple syrup was also evaluated. After only 20 minutes (duration of the analysis), its TSI value (TSI = 2.63) was about 8 times higher than the ones of the Ora-Blend oral liquids (Fig 2B).

### Analytical method validation

Regression analysis of the peak area of clonidine hydrochloride versus the concentration of each standard prepared from powder demonstrated linearity over the range of tested concentrations, with a coefficient of determination (R^2^) of 0.9992. Intra-day coefficient of variation was less than 0.50% and inter-day coefficient of variation was less than 2.00% for all concentrations. The accuracy of the method was calculated by comparing the regressed concentration to the know concentration of each point of the calibration curve. Errors of less than 0.60% were found for all concentrations.

**Fig 3:**
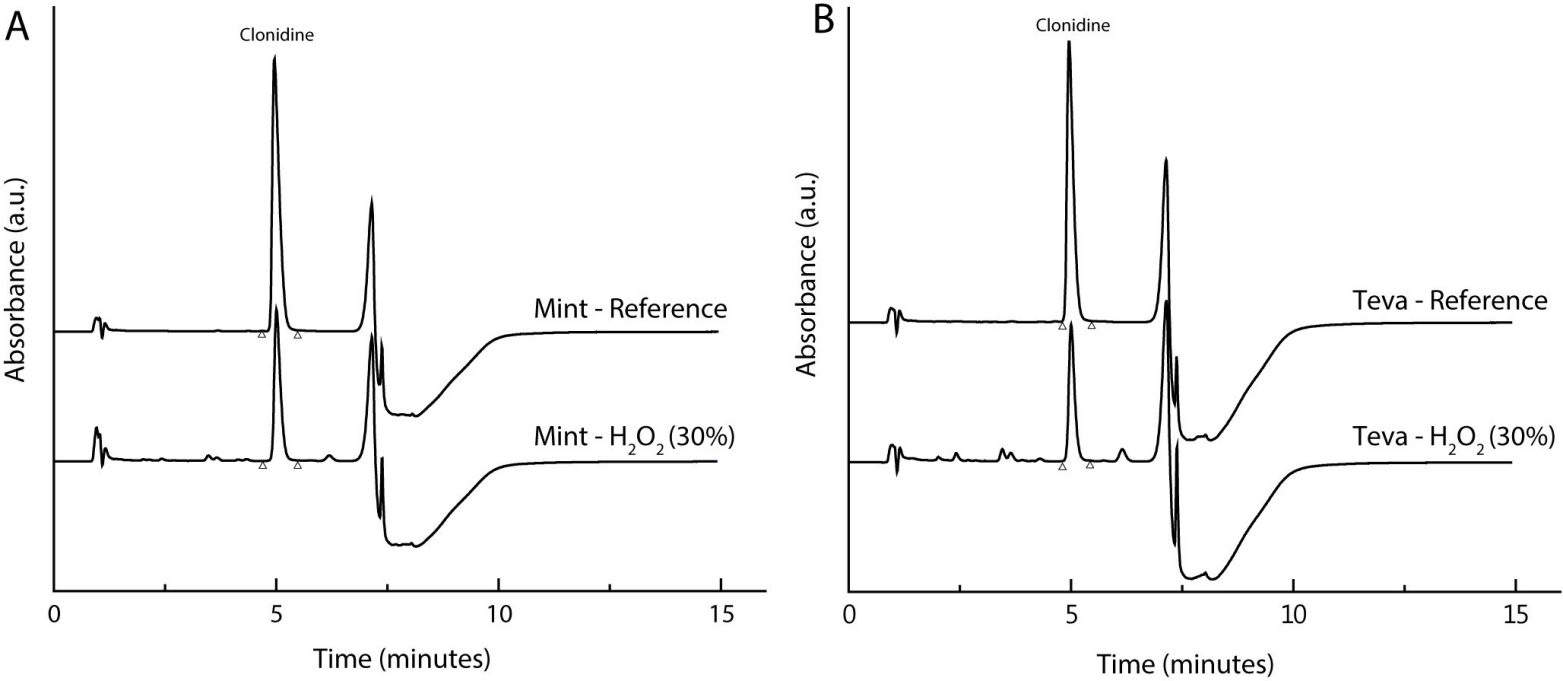
Representative chromatograms of clonidine hydrochloride degradation in oral liquids under extensive oxidizing conditions. Clonidine hydrochloride degradation under oxidative conditions with hydrogen peroxide (H_2_O_2_ 30%) for Mint (A) and Teva (B) oral liquids after 24 h at 60°C compared to their respective references.

Little or no degradation (less than 10%) was observed using hydrolytic (H_2_O), acidic (10 N, HCl) and alkaline (10 N, NaOH) conditions after 4h at 60°C on a preliminary study (S1 Table). Only oxidizing condition (30%, H_2_O_2_) was found to significantly degrade clonidine hydrochloride. After 24h at 60°C, about 50% of clonidine hydrochloride was degraded for both tablet brands (S2 Table). No degradation product interfered with the peak of clonidine hydrochloride, the purity of the peak was still 1.0000, which demonstrates the specificity of our analytical method (Fig 3).

### Chemical stability evaluation

**Fig 4:**
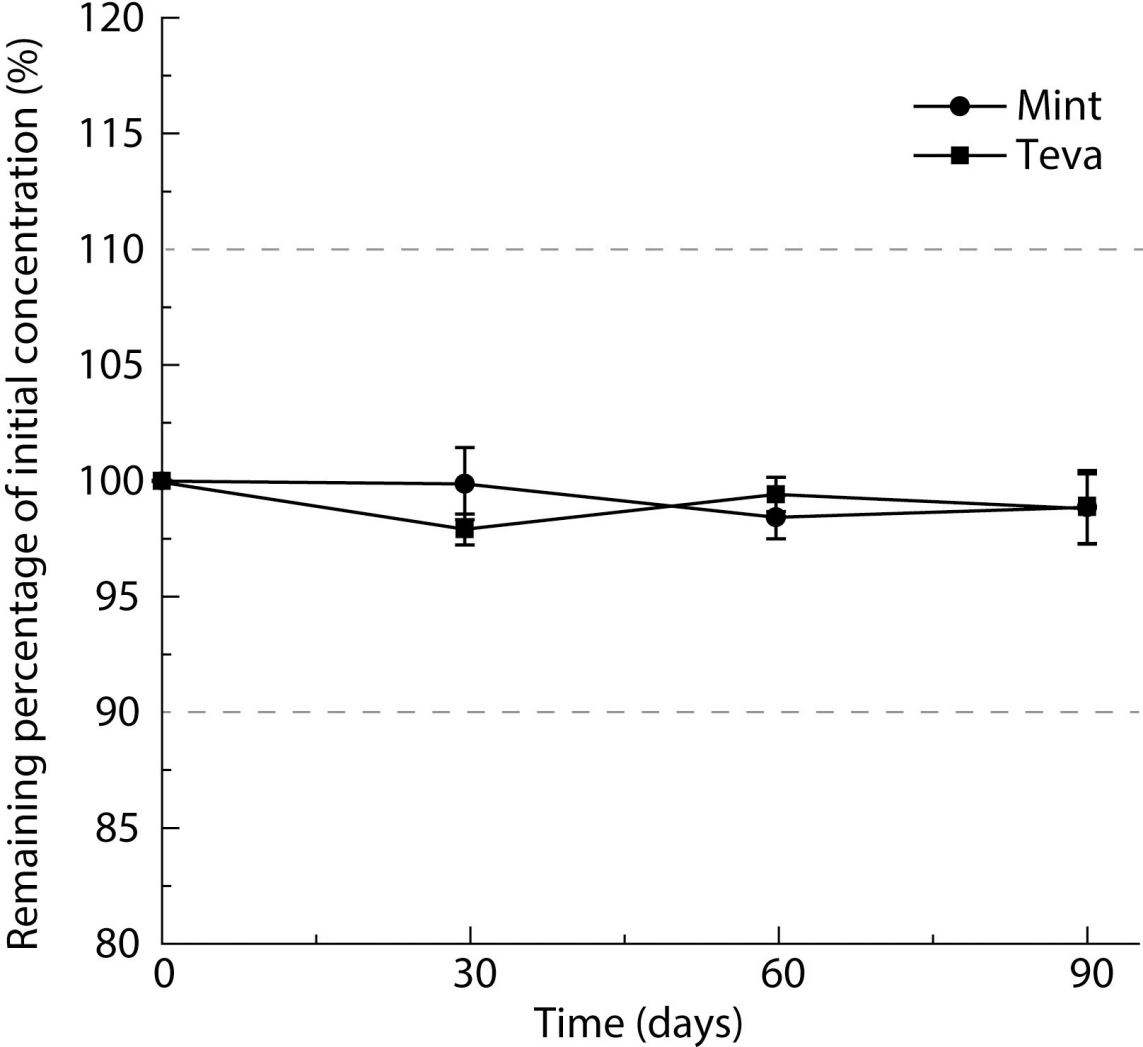
Stability of clonidine hydrochloride 20 μg/mL oral liquids in Ora-Blend at 25°C. Mean ± SD (correspond to standard deviation of measurements from three different bottles).

The pH of Teva and Mint oral liquids on day 0 were respectively 4.71 ± 0.03 and 4.56 ± 0.00. The mean pH of both oral liquids did not change by more than 0.01 over the entire study (S3 Table). The initial concentrations of oral liquids were respectively 100.0 ± 1.0% and 103.3 ± 0.7% of the target concentration (20 μg/mL) for Mint and Teva oral liquids. At all time points, the remaining percentage of the initial concentration was not less than 90.0% (Fig 4).

## Discussion

Compounding an oral liquid can be achieved by two ways; one consists in using a drug bulk powder, while the other lies in the grinding of commercial tablets. The first solution is probably the most adequate one for pediatric use as it limits the number of ingredients introduced in the final compounded formulation. However, it is often more practical and accessible for hospital and community pharmacists to use commercial tablets or capsules to prepare extemporaneous liquid dosage forms. Therefore, the excipients added to the formulation complicate its compounding and stability studies. In our case, we chose to formulate the 20- μg/mL clonidine oral liquid from two generic tablets, so as to offer a versatile formula to pharmacists. The resulting oral liquids encompass, in addition to clonidine hydrochloride, multiple substances [21, 22] (S2 Table). In order to quantify clonidine, the analytical procedure must either include a step to get rid of them or ensure proper separation without interference.

Conventional HPLC sample preparation for oral liquids usually consists in a liquid-liquid extraction, *i*.*e*. mixing the formulation with an appropriate solvent in order to precipitate excipients and solubilize the molecule of interest. The mixture is then centrifuged to ensure total separation of the pellet and the supernatant, containing the analyte. The supernatant will be free of particles and not viscous, which limits the damage that could be caused to the instrumentation. The formulation:solvent volume ratio is determined depending on the initial concentration of the formulation. Dilution of the formulation results in a decrease of the analyte concentration. The closer the analyte concentration to the detection limit, the greater the impacts of small variations on linearity and precision of the method.

**Fig 5:**
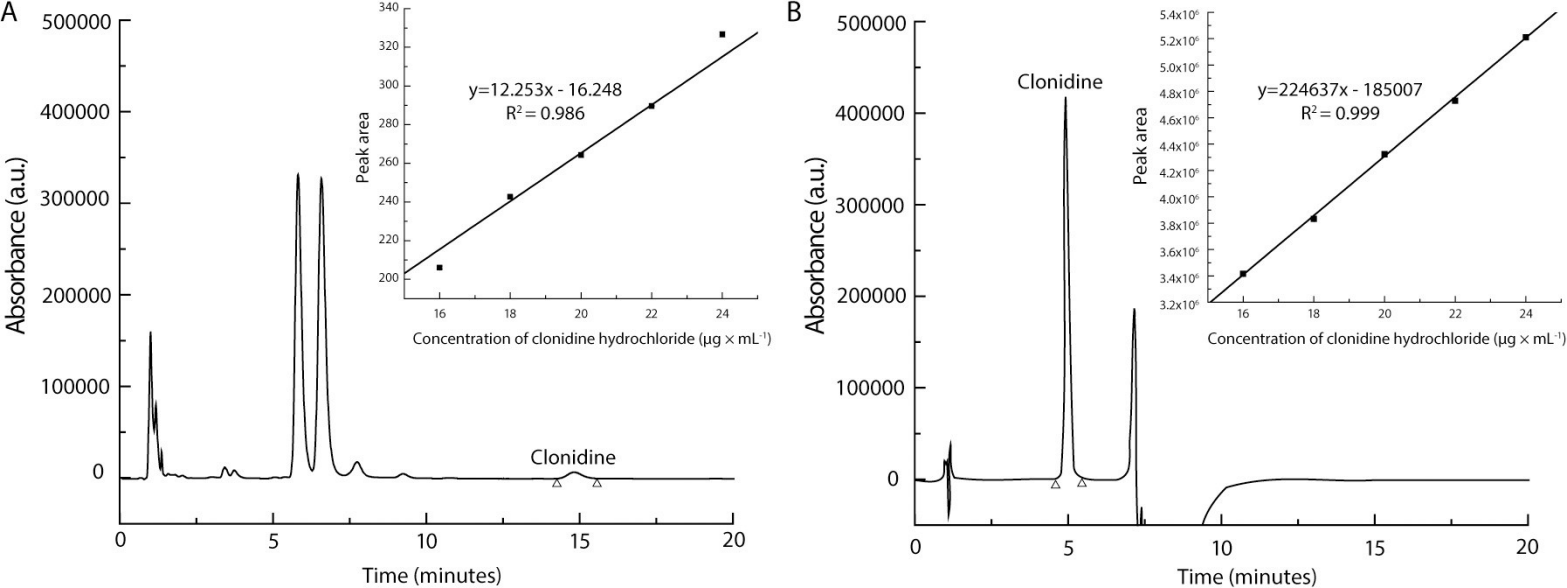
Evolution of the chromatographic analysis and its improvement over the quantification of clonidine hydrochloride. Chromatograms and calibration curves (inset) obtained (A) without SPE and (B) with SPE.

In this article, a clonidine oral liquid concentrated at 20 μg/mL has been studied. In our case, liquid-liquid extraction was not an acceptable option. Indeed, the low concentration of the oral liquid limited the volume of solvent which could be added during sample preparation in order to ensure an adequate analyte detection. A dilution ratio of 1:3 (formulation: methanol) resulted in a viscous and cloudy supernatant. The presence of many residual excipients from both tablets and Ora-Blend was confirmed by HPLC (Fig 5A). The method used for the analysis was the same as presented in "HPLC-UV method" section, except that the mobile phase was 80% A and 20% B in isocratic mode. Improvements of the analytical method were required to ensure efficient peak separation. The signal of clonidine hydrochloride was weak and many validation parameters, such as linearity (inset Fig 5A) and reproducibility, were not fulfilled, which resulted in the interruption of the analytical method development.

At this point, orienting the development towards an extraction method, such as solid phase extraction (SPE) seemed an interesting option to both purify and concentrate the analyte. SPE is a method normally used in bioanalysis when complex matrices are studied. Herein, the goal was to reduce excipient presence and sample viscosity before HPLC analysis. To the best of our knowledge, it is the first time that such an approach is used in drug oral liquid analysis. The absence of excipient peaks enabled the optimization of the HPLC method and resulted in shortening the analyte elution time (from 15 minutes to 5 minutes) (Fig 5). Calibration curve linearity was confirmed with a determination coefficient (R^2^) of 0.999 (inset Fig 5B). The most important improvement observed from this sample preparation was the peak area that increased by approximatively 15,000-fold.

Even though the sample preparation presented in this article is complex and involves a multi-step preparation, results and variations observed are within the acceptable range for the stability evaluation of compounded preparation [23–27]. The advantage of this method is the opportunity to concentrate the analyte to facilitate its detection and to drastically reduce the quantity of excipients injected in the HPLC system.

In parallel to the evaluation of the chemical stability, the physical stability must also be established. This often-overlooked aspect of the characterization of compounded oral liquids is nonetheless of prime importance. Indeed, the ease of resuspension as well as the homogeneity of the resuspensded liquid both warrant the delivery of a precise dose. In pediatrics, and especially with narrow therapeutic index drugs such as clonidine, as patients pertain to a fragile population, this aspect is even more crucial [13–16]. To assess this aspect, the common practice is grounded on sense-based observations: a visual inspection enables to check for color or fluidity changes, as well as presence of lumps, whereas an olfactive inspection can allow detecting smell changes. All these changes may reflect on instability or degradation of one or more of the oral liquid ingredients. This also enables to verify the resuspension process and ascertain that it is possible in everyday-life conditions. In this study, no changes were noted throughout the study, in terms of either color, smell or flow behaviour. Even if qualitative measures can be sufficient in some cases, encouraging the use of quantitative techniques could significantly improve the assessment of the physical stability and the detection of potential safety issues.

For this reason, static multiple light scattering (Turbiscan) was used to quantitively measure dispersion changes in these oral compounded oral liquids. This approach is quite recent and very few reports of such drug oral liquid analysis can be found. Nevertheless, its use is widely-spread, and acknowledged, in cosmetics and food industries [28–31]. The Turbiscan Stability Index (TSI), based on variations of transmittance and backscattering signals, was the parameter used to evaluate the physical stability of the different suspensions. The higher the TSI, or increasing over time, the poorest the stability of the suspension. Perturbations such as precipitation, creaming, aggregation can be detected, even at the nanoscale, depending on the perturbated areas on the graph [19]. From our observations, the oral liquids prepared in Ora-Blend were much more stable than the ones prepared in simple syrup. No oral liquids prepared in Ora-Blend displayed physical deterioration; dispersion profiles on day 0 and day 90 were similar. The choice of generic tablet did not seem to affect the physical stability of the oral liquid, which is not entirely unexpected as both tablet lists of ingredients display many common substances (S4 Table).

Thus, both qualitative and quantitative observations concurred to demonstrate that a 20-μg/mL clonidine oral liquid, compounded from two different generic tablets, was physically stable and that its physical stability would not affect the beyond-use date determination.

Comparing the pH of oral liquids on day 0 and day 90 showed no significant change. On the day of preparation (T0), all oral liquids complied with 100.0 ± 5.0% of the target concentration, indicating an easy compounding of oral liquids of the desired concentration. In the case of clonidine hydrochloride intended for pediatric patients, concentration accuracy is even more important because of its narrow therapeutic window, as already mentioned. The current regulations require that a compounded drug concentration remain in the ± 10.0% of the initial concentration to be considered safe to use [32]. In our case, at all time points, the mean concentration for both generic tablets remain largely above 90.0% of the initial concentration and no degradation peak appeared, demonstrating that those oral liquids were chemically stable through the entire study. Furthermore, the variability was no more than 5.0% of the initial concentration, which increases confidence of using this narrow therapeutic index compounded drug in neonates and small children. These results are not entirely surprising since clonidine hydrochloride was stable for the same duration when compounded from 100-μg tablets (Boehringer Ingelheim) at a concentration of 10 μg/mL in Ora-Blend [9].

## Conclusion

Clonidine hydrochloride was efficiently compounded as 20-μg/mL oral liquids prepared from two generic tablets (Teva and Mint) in Ora-Blend. Chemical stability was demonstrated using an original procedure, including SPE followed by a stability-indicating HPLC-UV method. Physical stability over a period of 90 days was observed by the conventional visual inspections and confirmed by an optical quantification performed through static multiple light scattering. This approach enabled to evidence that the physical instability issues reported for simple syrup were no longer observed with Ora-Blend. Clonidine hydrochloride concentrations remained in the 95.0–105.0% range of initial concentration during the whole study. From these results, a beyond-use date of 90 days can be assigned to 20-μg/mL clonidine hydrochloride oral liquids in Ora-Blend stored in amber plastic bottles at 25°C.

## Supporting information

S1 Table Degradation of clonidine hydrochloride oral liquids under stressing conditions. (PDF)

S2 Table Degradation of clonidine hydrochloride oral liquids under extensive oxidizing conditions. (PDF)

S3 Table pH measurements. Report of initial (T0) and final (T90) pH of clonidine hydrochloride oral liquids.
(PDF)

S4 Table List of non-medicinal ingredients in 0.1 mg clonidine hydrochloride tablets from Mint and Teva generic tablets. (PDF)

S1 Appendix HPLC results. Excel file containing all reported HPLC results.
(XLSX)

S2 Appendix HPLC results analysis. Excel file containing all analysis performed from HPLC results.
(XLSX)

S3 Appendix Turbiscan results and chromatograms. (ZIP)
