# Supplementary material for: Stability evaluation of compounded clonidine hydrochloride oral liquids based on a solid-phase extraction HPLC-UV method
Source: PLoS One. 2021 Nov 30;16(11):e0260279. doi: 10.1371/journal.pone.0260279 (PMC8631633; doi:10.1371/journal.pone.0260279)
Supplement: S1 Table — (PDF) [file pone.0260279.s001.pdf]

Mint			Teva		
Condition	Peak area	% of the reference	Condition	Peak area	% of the reference
Reference (H_2O, 4°C)	2902189	-------	Reference (H_2O, 4°C)	2847613	-------
Hydrolytic (H_2O, 60°C)	2848234	98.14	Hydrolytic (H_2O, 60°C)	2842686	99.83
Oxidative (30% H_2O_2, 60°C)	2435919	83.93	Oxidative (30% H_2O_2, 60°C)	2527768	88.77
Acidic (10 N HCl, 60°C)	2664998	91.83	Acidic (10 N HCl, 60°C)	2635586	92.55
Basic (10 N HCl, 60°C)	2794373	96.29	Basic (10 N HCl, 60°C)	2734189	96.02
