# Supplementary material for: Stability evaluation of compounded clonidine hydrochloride oral liquids based on a solid-phase extraction HPLC-UV method
Source: PLoS One. 2021 Nov 30;16(11):e0260279. doi: 10.1371/journal.pone.0260279 (PMC8631633; doi:10.1371/journal.pone.0260279)
Supplement: S2 Table — (PDF) [file pone.0260279.s002.pdf]

Mint			Teva		
Condition	Peak area	% of the reference	Condition	Peak area	% of the reference
Reference (H_2O, 4°C)	4552790	-------	Reference (H_2O, 4°C)	3142078	-------
Oxidative (30% H_2O_2, 60°C)	2241213	49.23	Oxidative (30% H_2O_2, 60°C)	1391310	44.28
