# Supplementary material for: Stability evaluation of compounded clonidine hydrochloride oral liquids based on a solid-phase extraction HPLC-UV method
Source: PLoS One. 2021 Nov 30;16(11):e0260279. doi: 10.1371/journal.pone.0260279 (PMC8631633; doi:10.1371/journal.pone.0260279)
Supplement: S3 Table — Report of initial (T0) and final (T90) pH of clonidine hydrochloride oral liquids. (PDF) [file pone.0260279.s003.pdf]

**Mint**			**Teva**		
Time point	pH	Mean ± SD	Time point	pH	Mean ± SD
T0	4.56	4.56 ± 0.00	T0	4.74	4.71 ± 0.03
	4.56			4.71	
	4.56			4.68	
T90	4.57	4.57 ± 0.01	T90	4.75	4.71 ± 0.04
	4.58			4.67	
	4.57			4.71	
