# Supplementary material for: Stability evaluation of compounded clonidine hydrochloride oral liquids based on a solid-phase extraction HPLC-UV method
Source: PLoS One. 2021 Nov 30;16(11):e0260279. doi: 10.1371/journal.pone.0260279 (PMC8631633; doi:10.1371/journal.pone.0260279)
Supplement: S4 Table — (PDF) [file pone.0260279.s004.pdf]

**Mint**	**Teva**
Colloidal silicon dioxide	Colloidal silicon dioxide
Dibasic calcium phosphate	Dibasic calcium phosphate
Magnesium stearate	Magnesium stearate
Lactose monohydrate	Lactose monohydrate (spray dried)
Corn starch dried	Pregelatinized starch.
FD&C Red #40	
Hydroxypropyl cellulose	
Microcrystalline cellulose	
