# Supplementary material for: Stability evaluation of compounded clonidine hydrochloride oral liquids based on a solid-phase extraction HPLC-UV method
Source: PLoS One. 2021 Nov 30;16(11):e0260279. doi: 10.1371/journal.pone.0260279 (PMC8631633; doi:10.1371/journal.pone.0260279)
Supplement: S3 Appendix — (ZIP) [file pone.0260279.s007.zip › S3_Appendix/S3_Appendix_OB_T0.pdf]

Turbiscan Analysis Report

Clonidine - T0 - 25C

Raw Data - T & BS

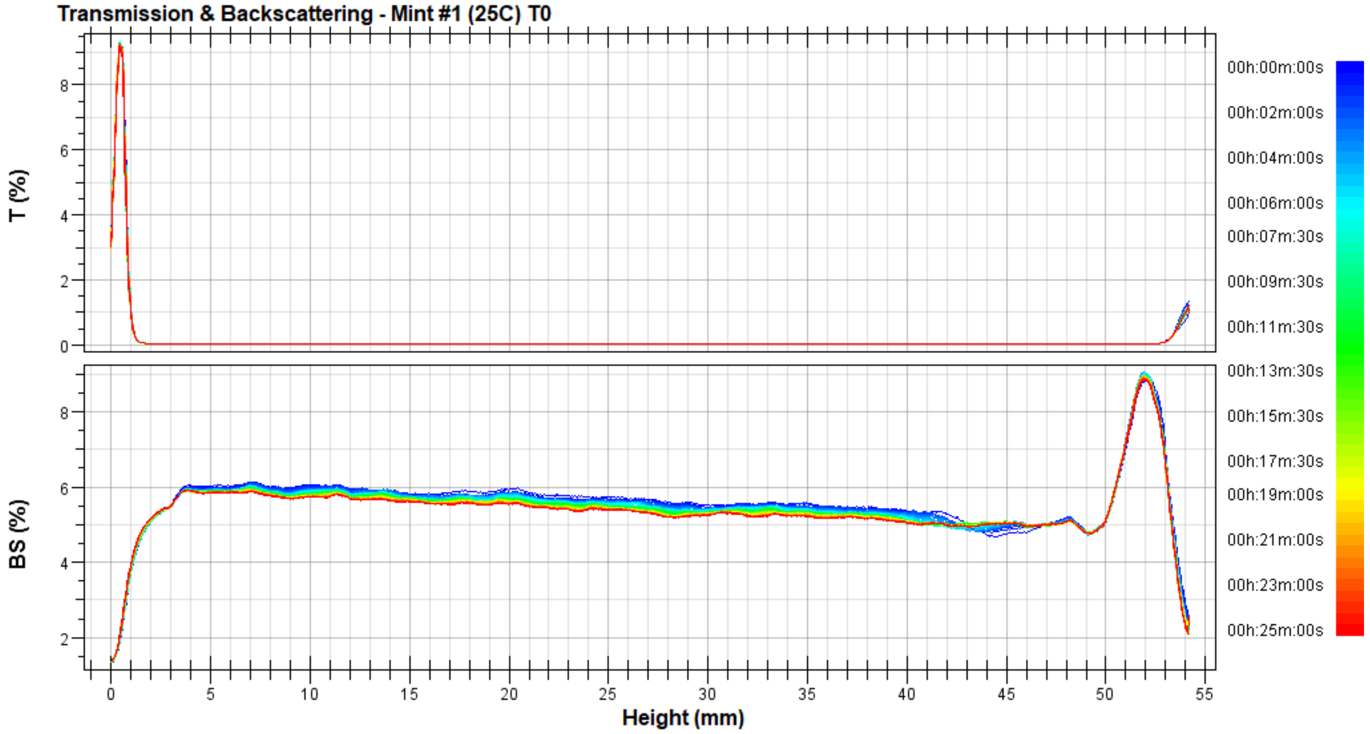

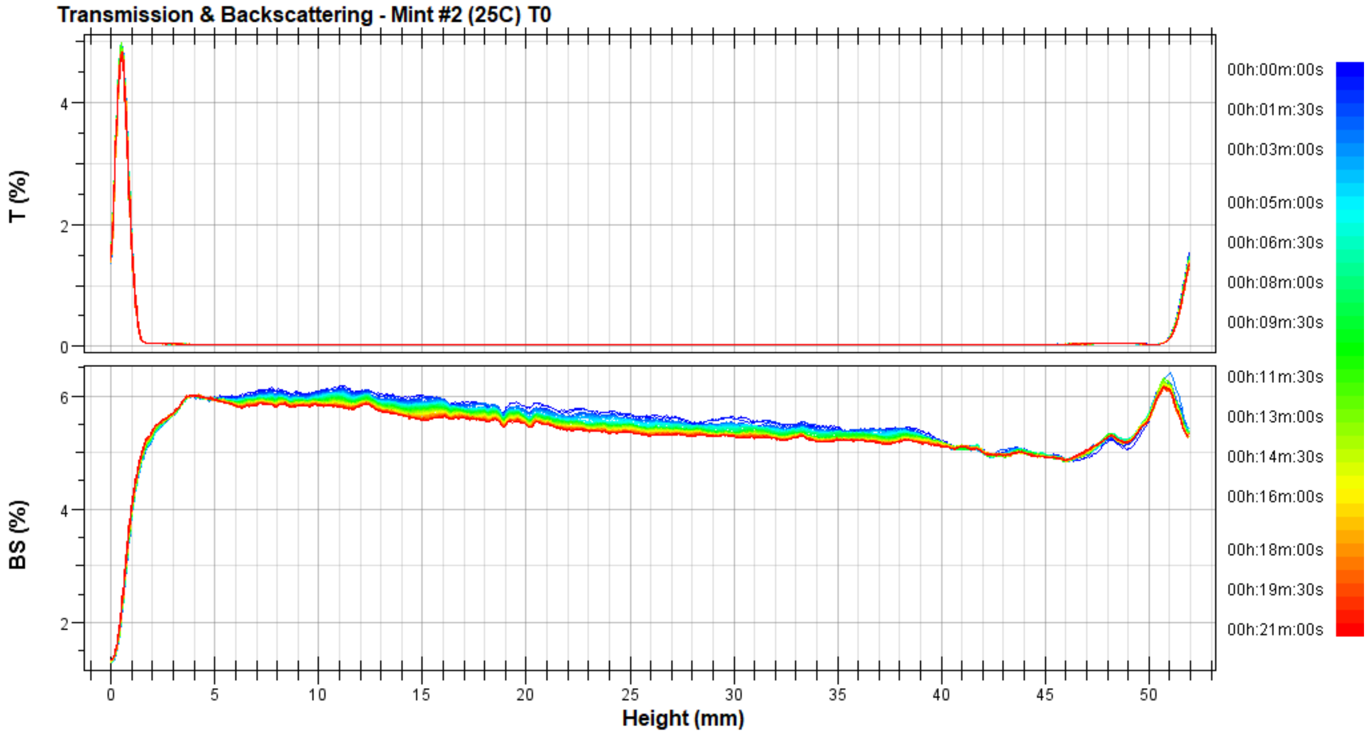

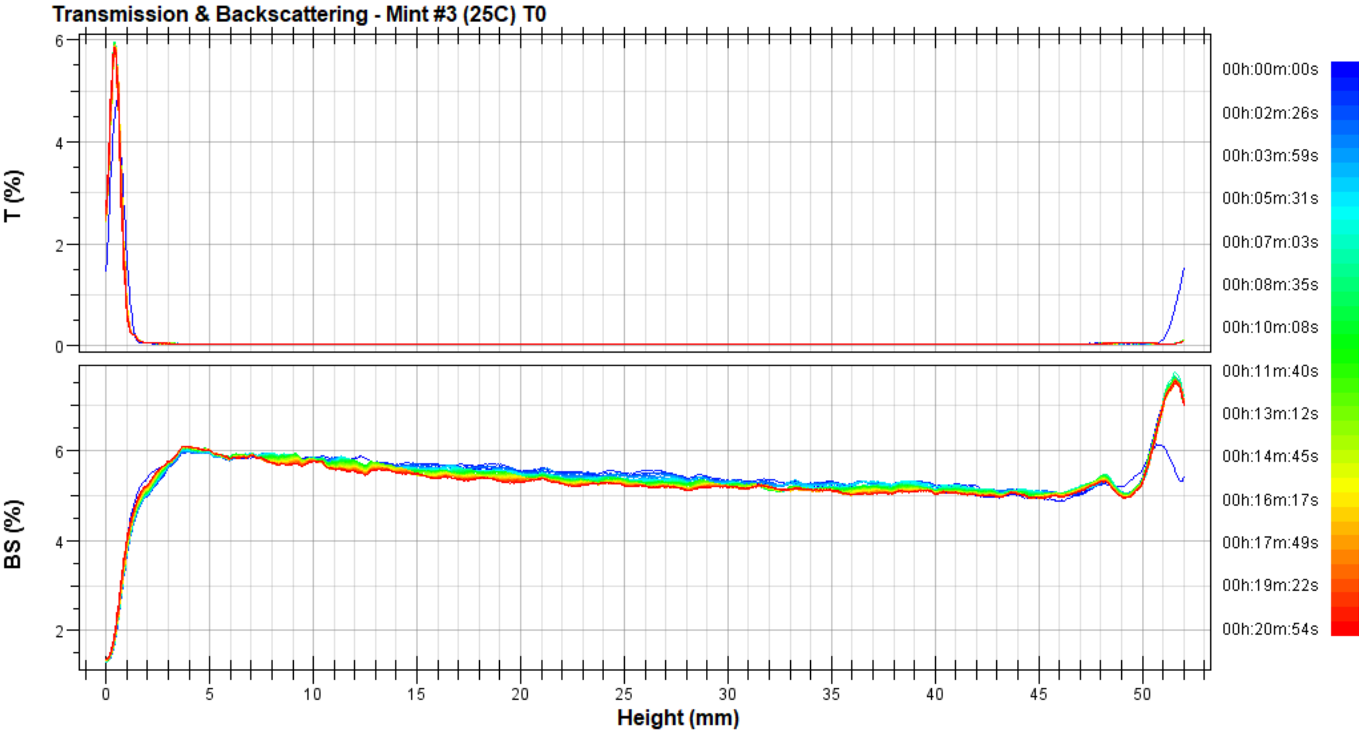
Transmission & Backscattering - Mint #3 (25C) T0
T (%)
BS (%)
Height (mm)
00h:00m:00s
00h:02m:26s
00h:03m:59s
00h:05m:31s
00h:07m:03s
00h:08m:35s
00h:10m:08s
00h:11m:40s
00h:13m:12s
00h:14m:45s
00h:16m:17s
00h:17m:49s
00h:19m:22s
00h:20m:54s

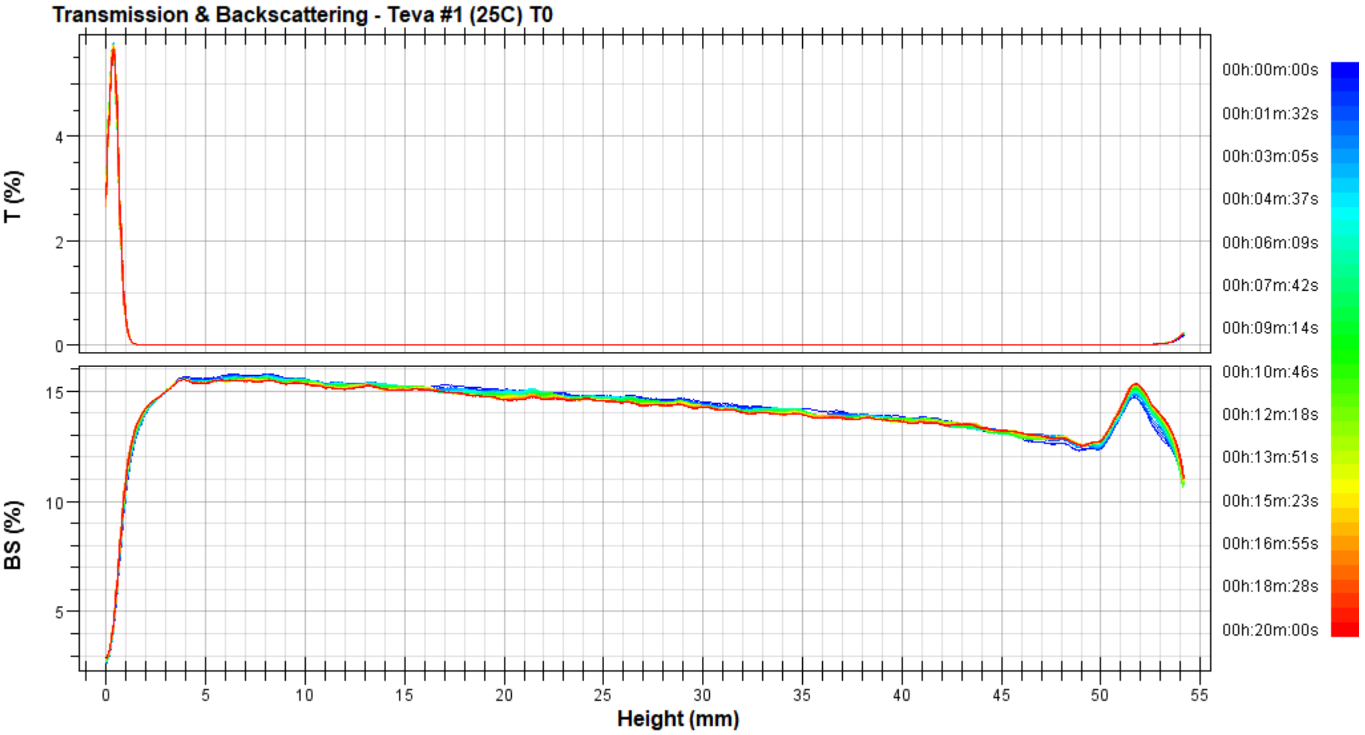
Transmission & Backscattering - Teva #1 (25C) T0
T (%)
BS (%)
Height (mm)
00h:00m:00s
00h:01m:32s
00h:03m:05s
00h:04m:37s
00h:06m:09s
00h:07m:42s
00h:09m:14s
00h:10m:46s
00h:12m:18s
00h:13m:51s
00h:15m:23s
00h:16m:55s
00h:18m:28s
00h:20m:00s

Transmission & Backscattering - Teva #1 (25C) T0

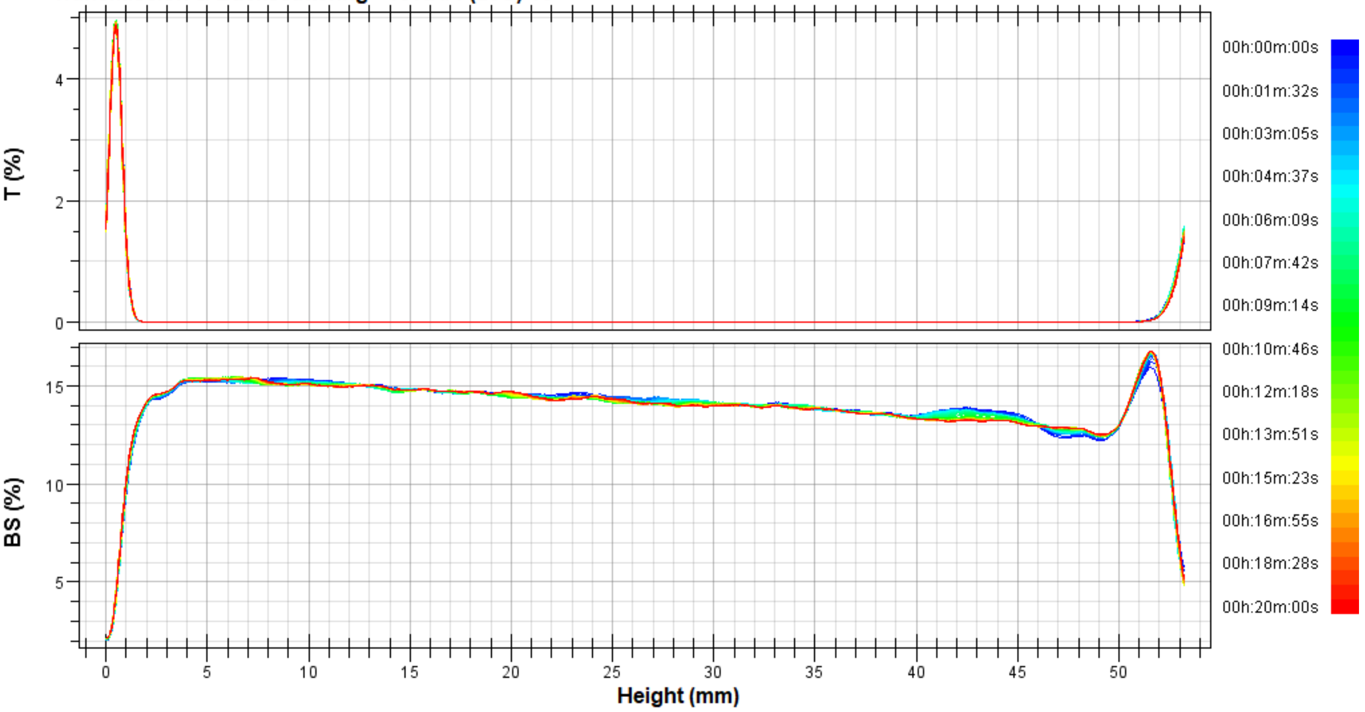

Transmission & Backscattering - Teva #1 (25C) T0

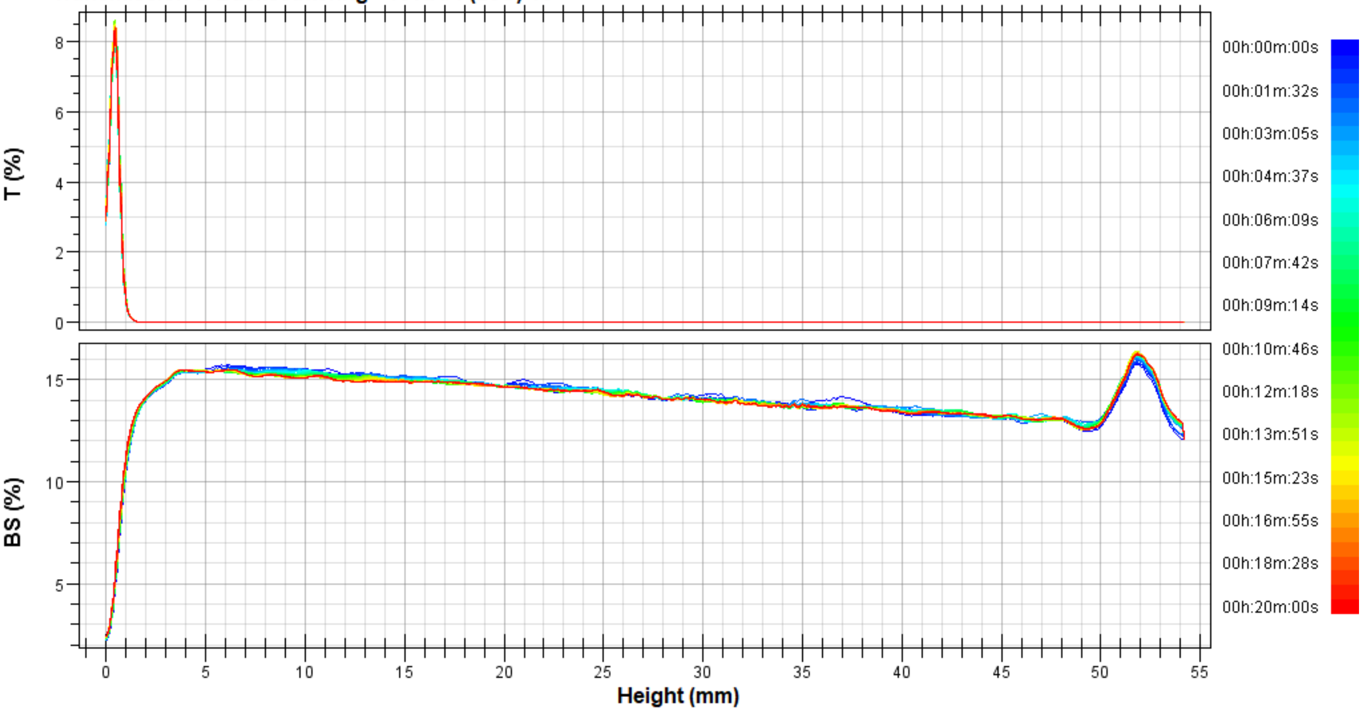

Destabilisation - TSI (global)

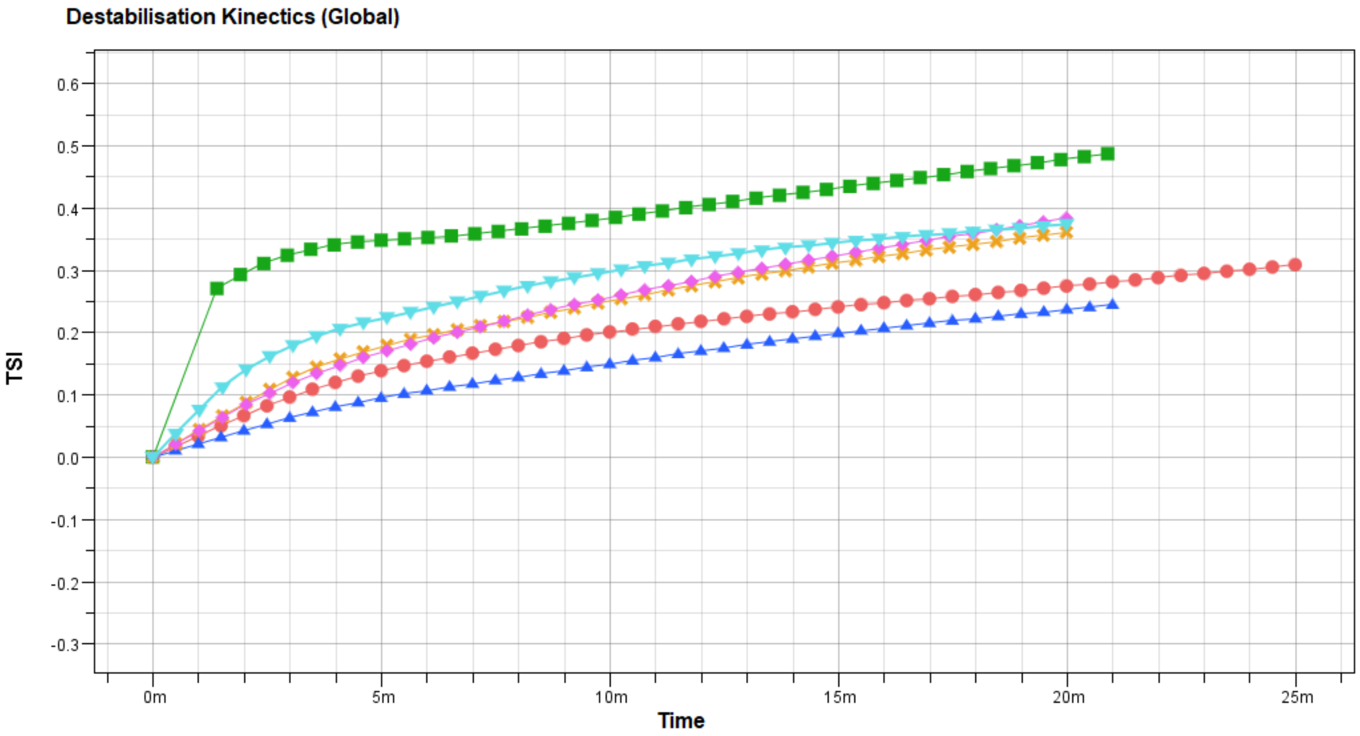

Graphic Tools - Data Table 1

Measurement	TSI (Global) 20mn
Mint #1 (25C) T0	0.3
Mint #2 (25C) T0	0.2
Mint #3 (25C) T0	0.5
Teva #1 (25C) T0	0.4
Teva #1 (25C) T0	0.4
Teva #1 (25C) T0	0.4
