# Supplementary material for: Stability evaluation of compounded clonidine hydrochloride oral liquids based on a solid-phase extraction HPLC-UV method
Source: PLoS One. 2021 Nov 30;16(11):e0260279. doi: 10.1371/journal.pone.0260279 (PMC8631633; doi:10.1371/journal.pone.0260279)
Supplement: S3 Appendix — (ZIP) [file pone.0260279.s007.zip › S3_Appendix/S3_Appendix_OB_T90.pdf]

Turbiscan Analysis Report

Clonidine - T90 - 25C

Raw Data - T & BS

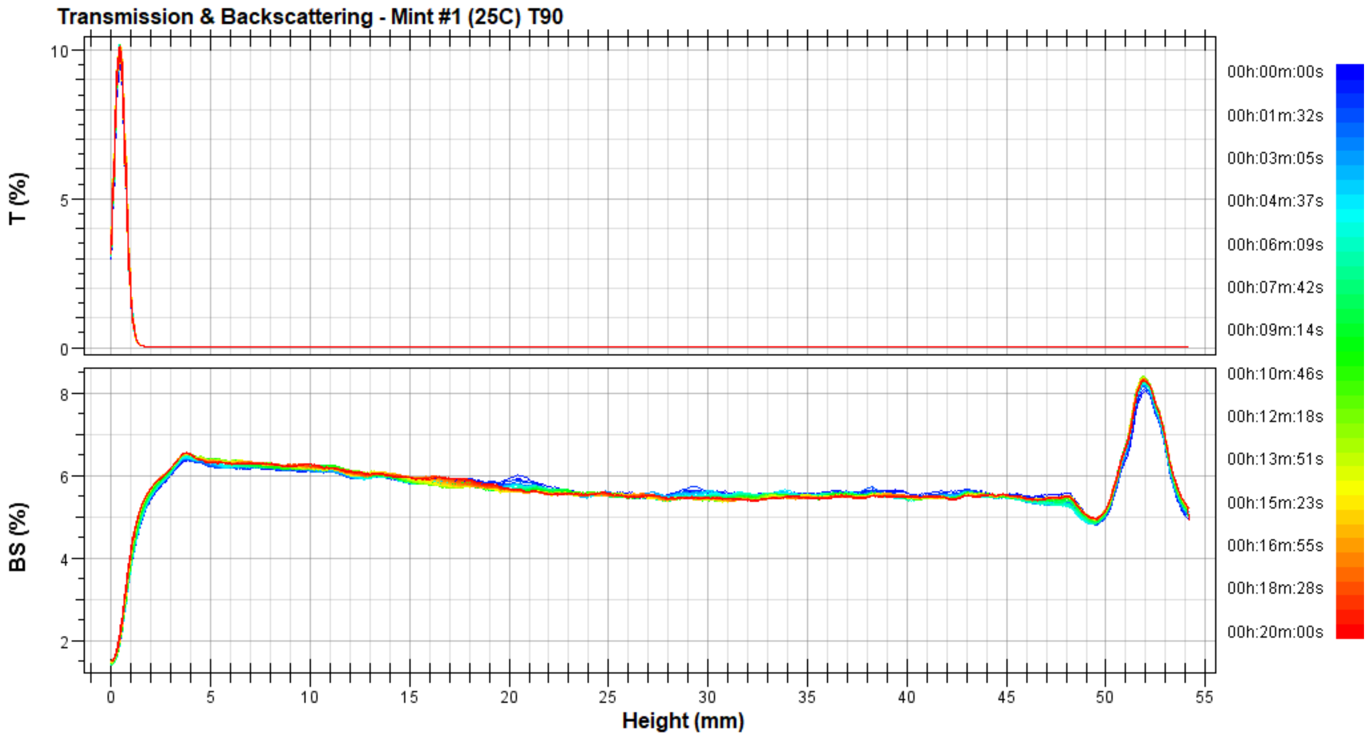

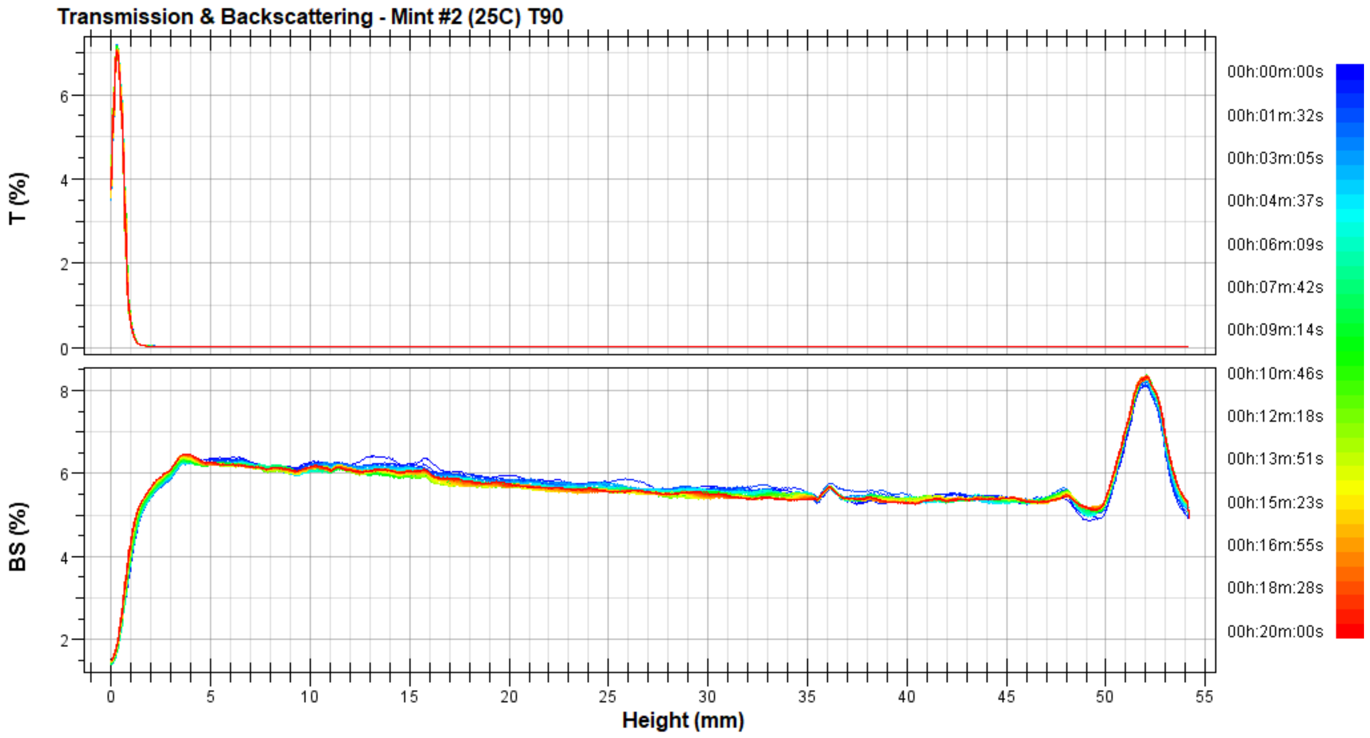

Transmission & Backscattering - Teva #1 (25C) T90

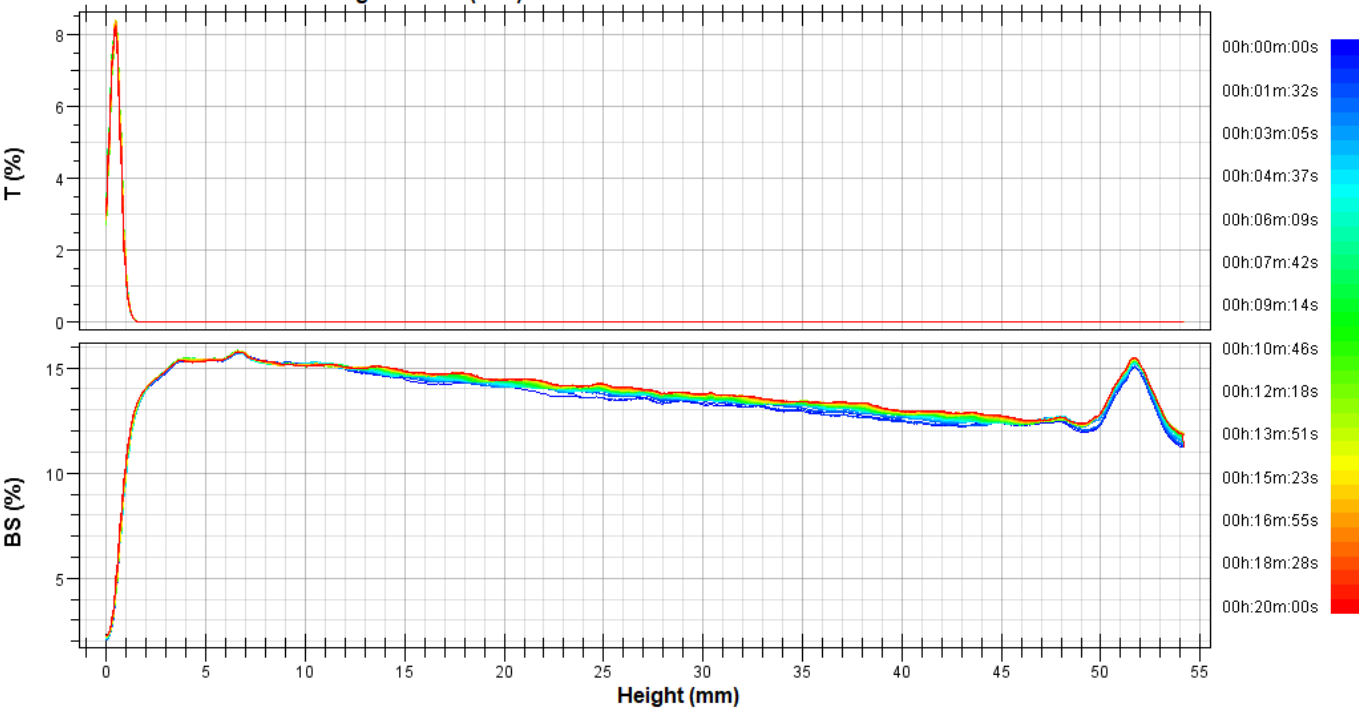

Transmission & Backscattering - Teva #2 (25C) T90

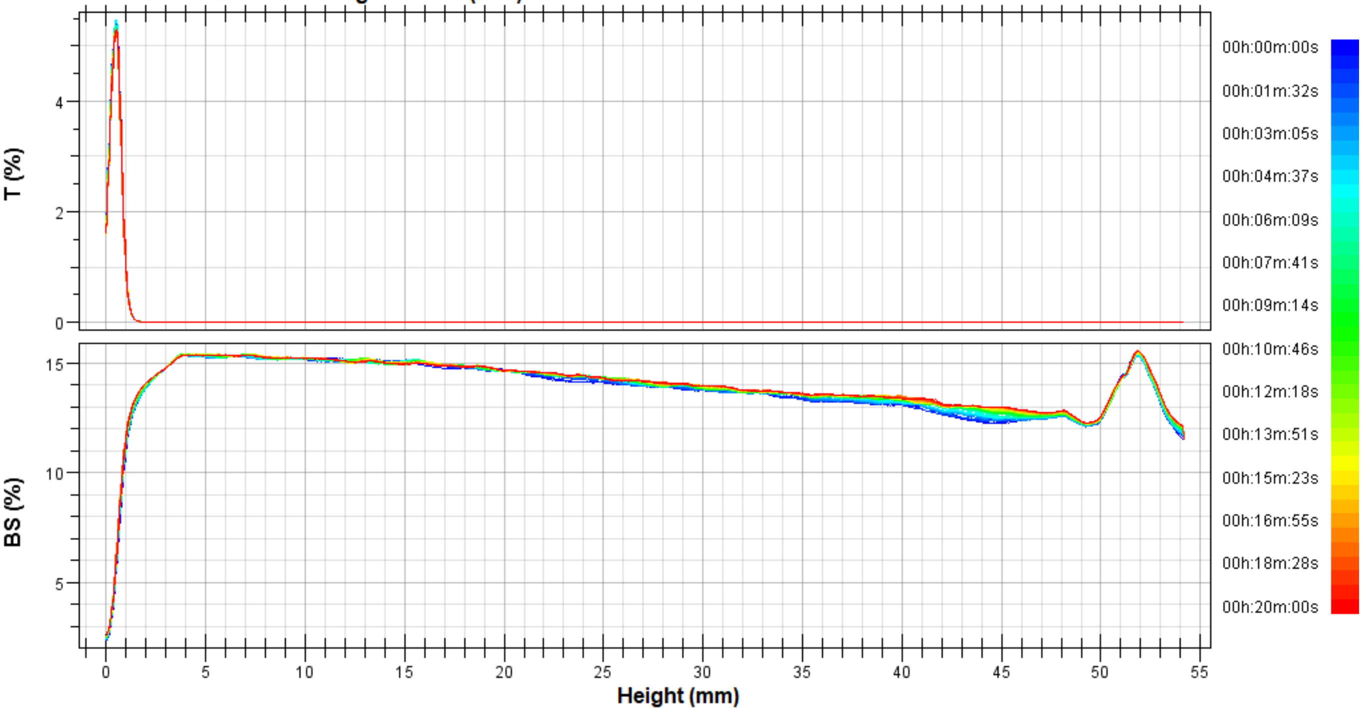

Transmission & Backscattering - Teva #3 (25C) T90

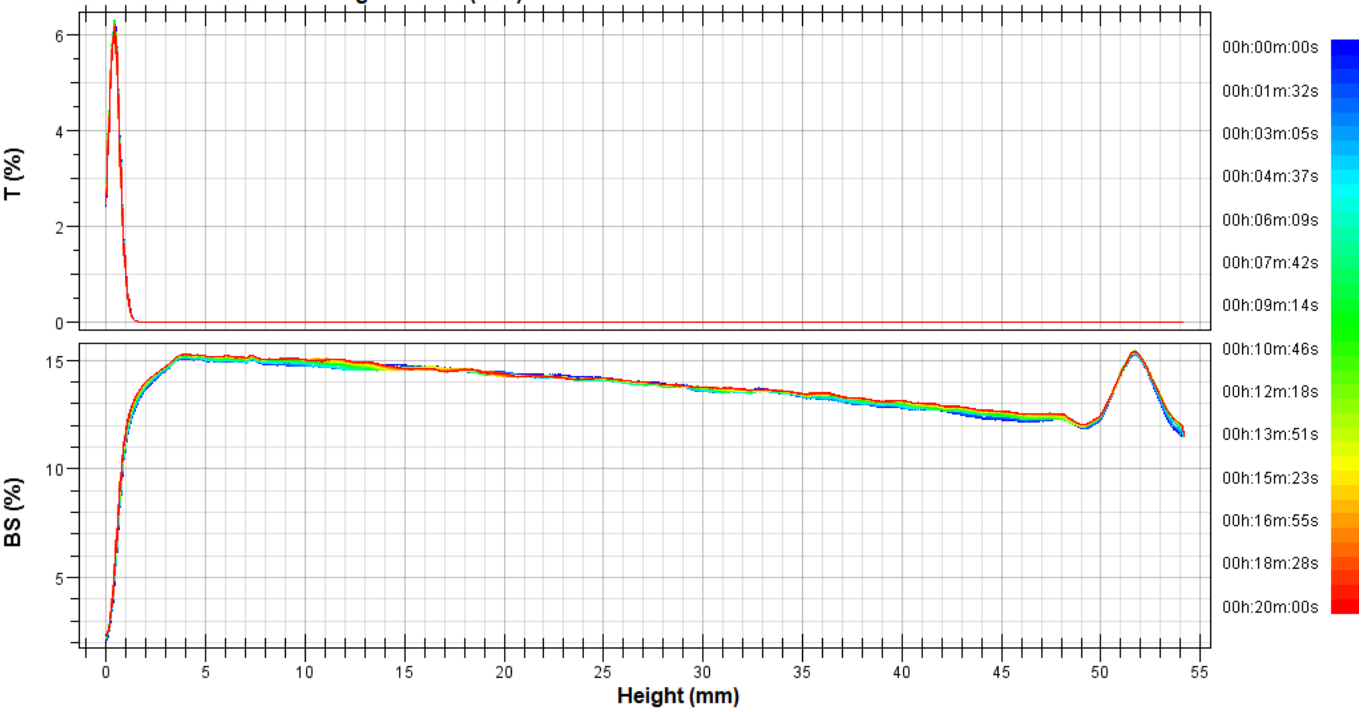

Transmission & Backscattering - Mint #3 (25C) T90

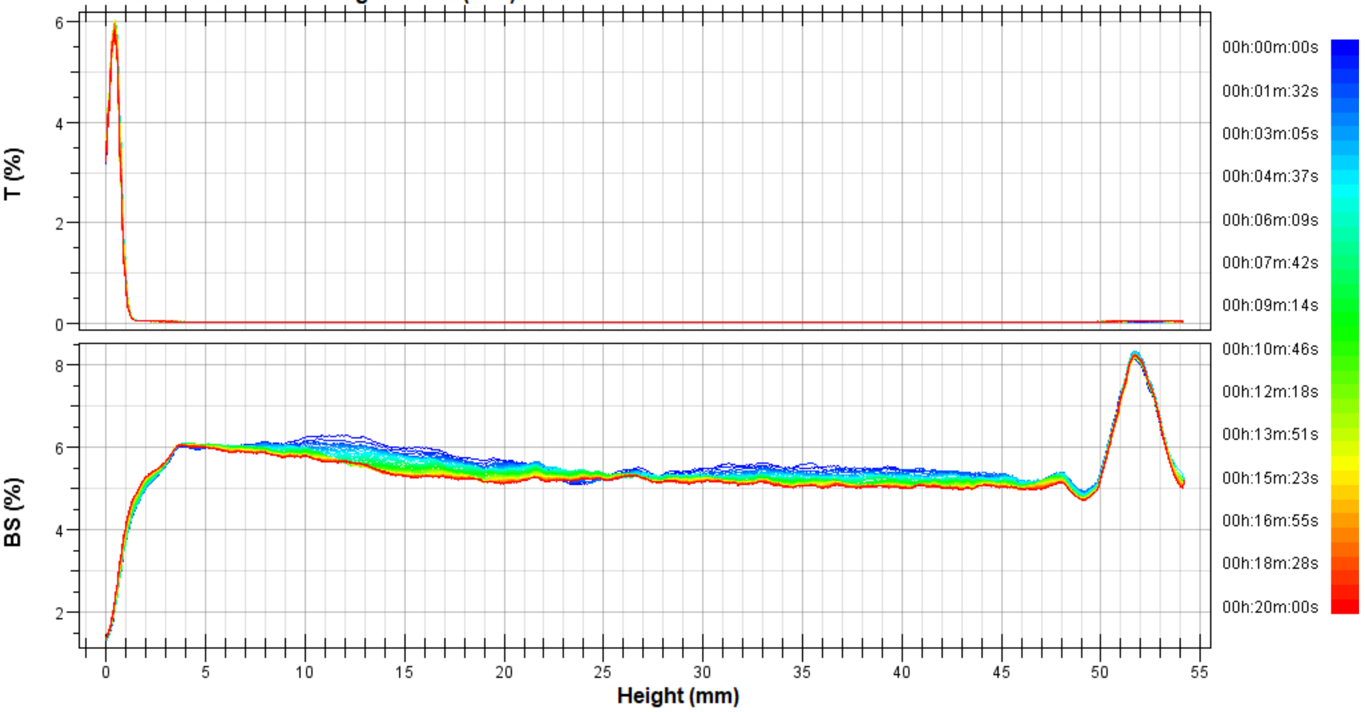

Destabilisation - TSI (global)

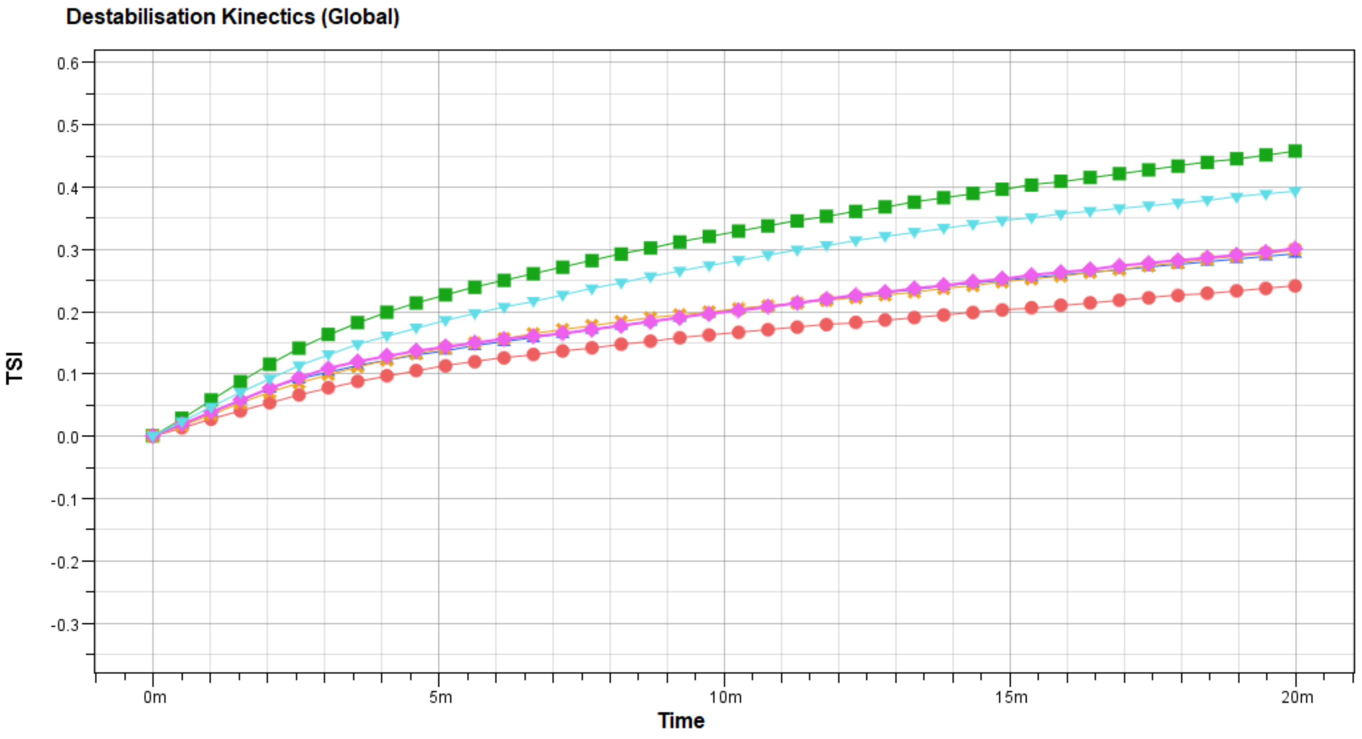

Graphic Tools - Data Table 1

Measurement	TSI (Global)<br>20mn
Mint #1 (25C) T90	0.2
Mint #2 (25C) T90	0.3
Mint #3 (25C) T90	0.4
Teva #1 (25C) T90	0.5
Teva #2 (25C) T90	0.3
Teva #3 (25C) T90	0.3
