# Supplementary material for: Stability evaluation of compounded clonidine hydrochloride oral liquids based on a solid-phase extraction HPLC-UV method
Source: PLoS One. 2021 Nov 30;16(11):e0260279. doi: 10.1371/journal.pone.0260279 (PMC8631633; doi:10.1371/journal.pone.0260279)
Supplement: S3 Appendix — (ZIP) [file pone.0260279.s007.zip › S3_Appendix/S3_Appendix_SS_T0.pdf]

Turbiscan Analysis Report

Sirop simple 1

Raw Data - T & BS

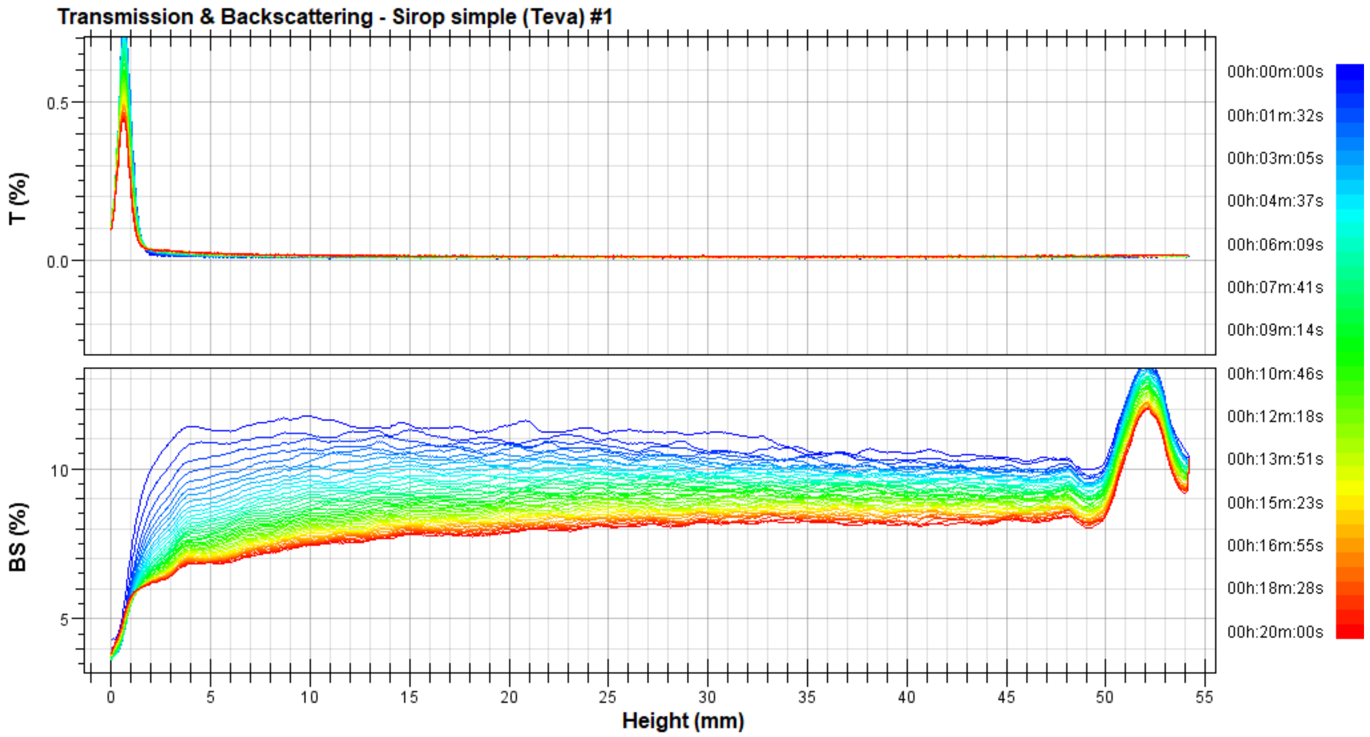

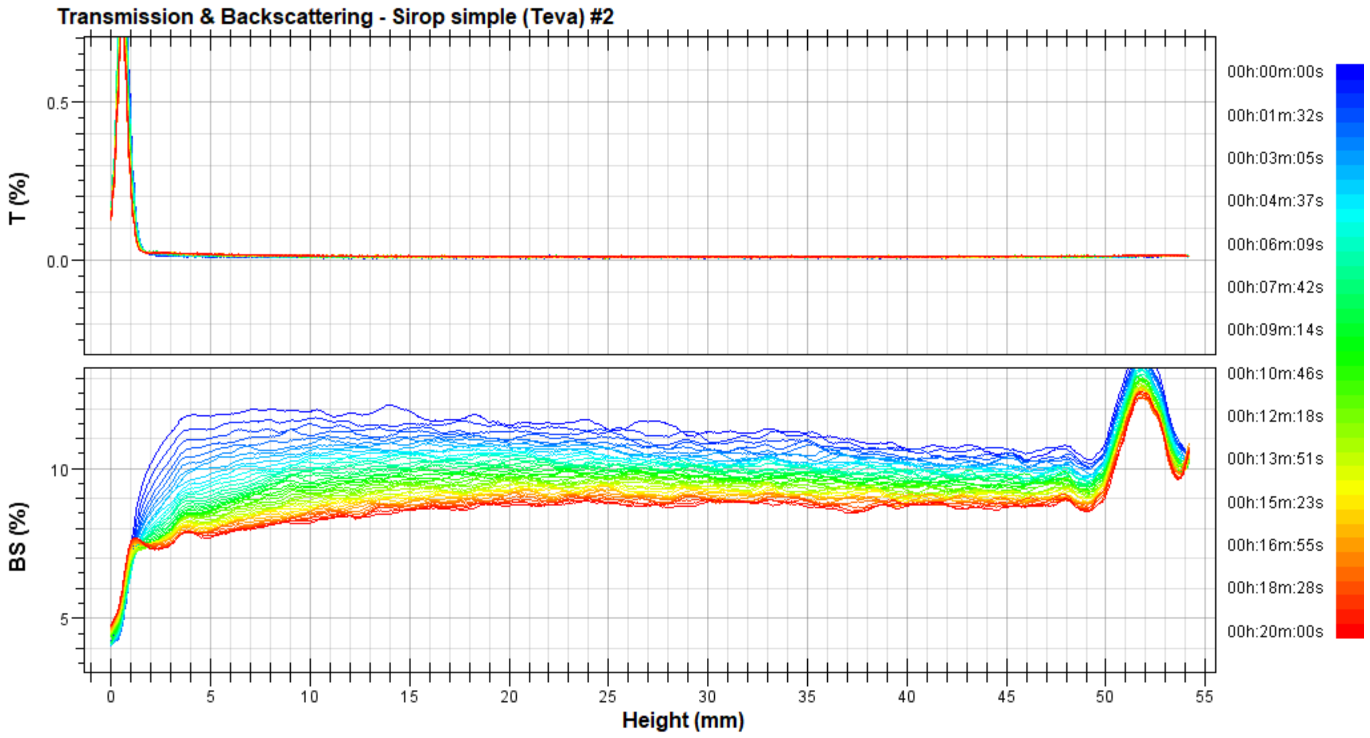

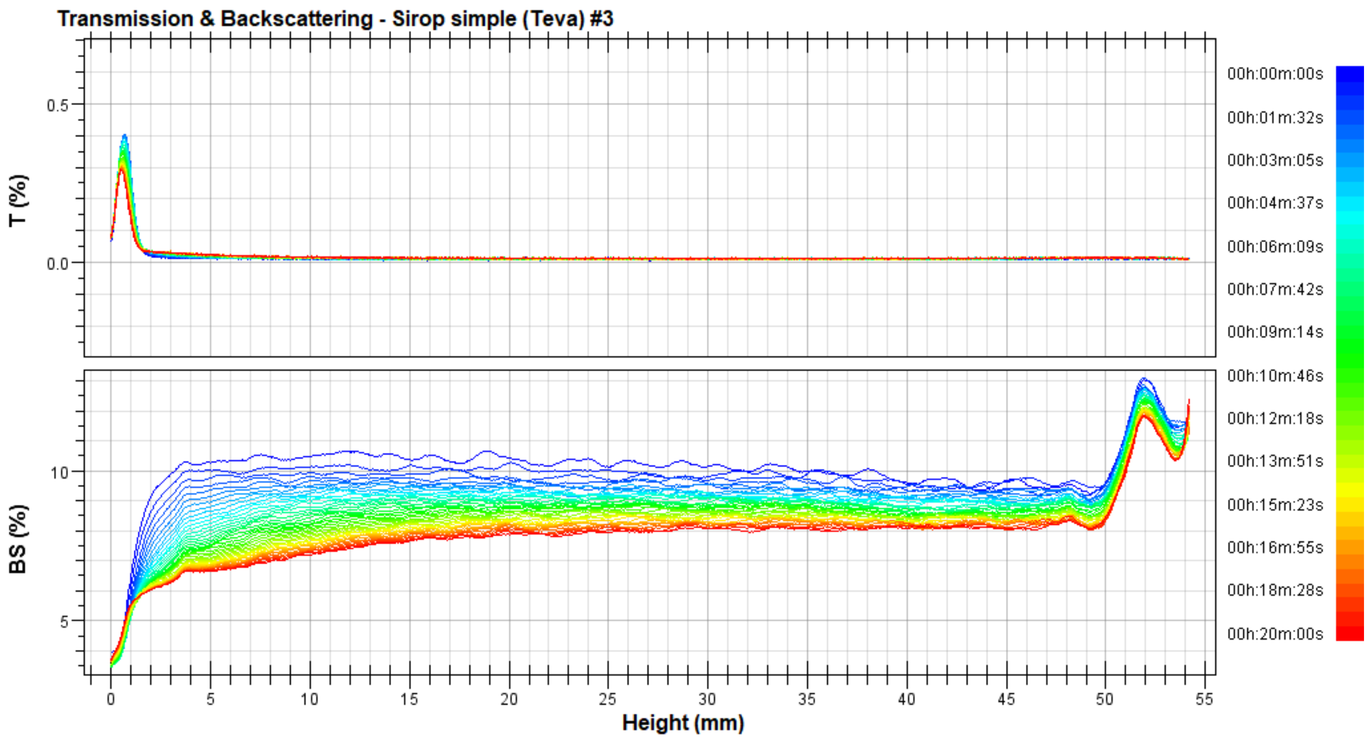

Destabilisation - TSI (global)

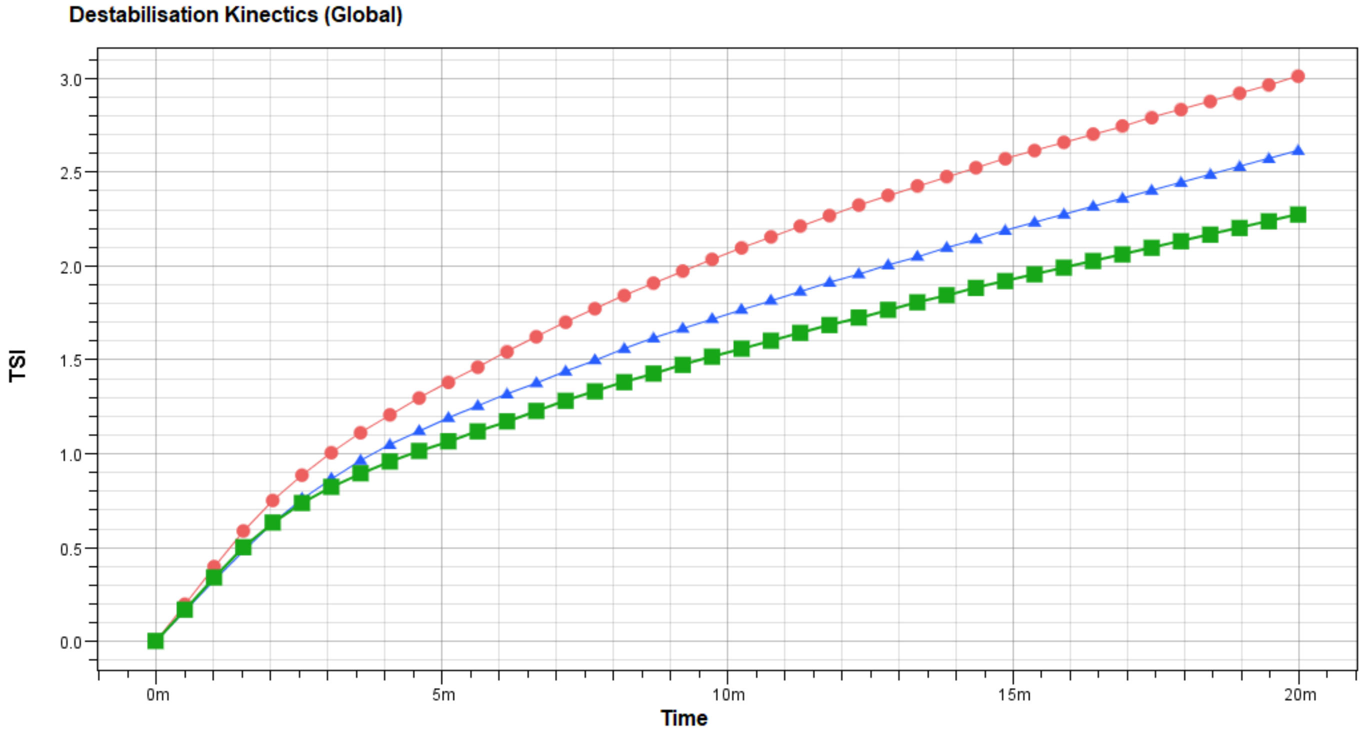

Graphic Tools - Data Table 1

Measurement	TSI (Global)<br>20mn
Sirop simple (Teva) #1	3.0
Sirop simple (Teva) #2	2.6
Sirop simple (Teva) #3	2.3
